# Variation in the Chemical Composition of Five Varieties of *Curcuma longa* Rhizome Essential Oils Cultivated in North Alabama

**DOI:** 10.3390/foods10020212

**Published:** 2021-01-21

**Authors:** William N. Setzer, Lam Duong, Ambika Poudel, Srinivasa Rao Mentreddy

**Affiliations:** 1 Department of Chemistry, University of Alabama in Huntsville, Huntsville, AL 35899, USA; 2 Aromatic Plant Research Center, 230 N 1200 E, Suite 100, Lehi, UT 84043, USA; apoudel@aromaticplant.org; 3 Department of Biological and Environmental Sciences, Alabama A&M University, Normal, AL 35762, USA; lamduongvn@gmail.com

**Keywords:** turmeric, essential oil composition, α-turmerone, β-turmerone, *ar*-turmerone

## Abstract

Turmeric (*Curcuma longa* L.) is an important spice, particularly is Asian cuisine, and is also used in traditional herbal medicine. Curcuminoids are the main bioactive agents in turmeric, but turmeric essential oils also contain health benefits. Turmeric is a tropical crop and is cultivated in warm humid environments worldwide. The southeastern United States also possesses a warm humid climate with a growing demand for locally sourced herbs and spices. In this study, five different varieties of *C. longa* were cultivated in north Alabama, the rhizome essential oils obtained by hydrodistillation, and the essential oils were analyzed by gas chromatographic techniques. The major components in the essential oils were α-phellandrene (3.7–11.8%), 1,8-cineole (2.6–11.7%), α-zingiberene (0.8–12.5%), β-sesquiphellandrene (0.7–8.0%), *ar*-turmerone (6.8–32.5%), α-turmerone (13.6–31.5%), and β-turmerone (4.8–18.4%). The essential oil yields and chemical profiles of several of the varieties are comparable with those from tropical regions, suggesting that these should be considered for cultivation and commercialization in the southeastern United States.

## 1. Introduction

**Figure 1:**
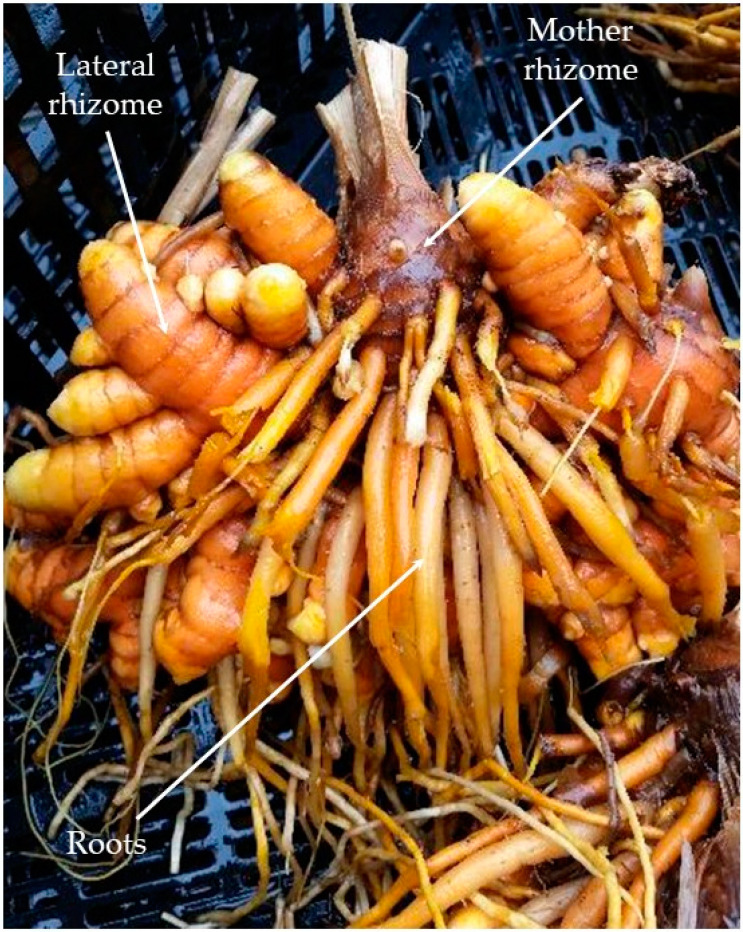
Underground parts of *Curcuma longa* showing the rhizomes and roots.

Turmeric *(Curcuma longa* L.), belonging to Zingiberaceae, is a rhizomatous plant native to Southeast Asia, but is extensively cultivated worldwide, particularly in tropical countries (e.g., India, Pakistan, Bangladesh, China, Taiwan, Thailand, Sri Lanka, East Indies, Burma, Indonesia, northern Australia, Costa Rica, Haiti, Jamaica, Peru, and Brazil) [1,2,3,4]. Turmeric is well known for its use as a culinary ingredient and a traditional herbal medicine [5]. It is extensively used in Asian cuisine and is one of the key ingredients in curry powders [6]. Turmeric, either fresh or in dried form, has a long history of medicinal use, dating back 4000 years [7]. Due to its bright yellow/orange color, turmeric is often referred to as the “Indian saffron” or “golden spice”. Curcuminoids are considered the main bioactive components of turmeric. Turmeric’s medical properties are credited mainly to the curcuminoids, which are abundant in turmeric rhizome [8]. The total curcumin (sum of all curcuminoids) was proven to have significant health benefits along with the potential to prevent various diseases, including Alzheimer’s, coronary heart disease, and cancer [9]. Due to a plethora of scientific articles on the health benefits of turmeric, the demand for turmeric is steadily increasing in the US and now represents an estimated US$36 million per annum. The US imports 90% of its requirements from various countries [10]. Due to recent inconsistencies associated with quality and production methods of raw materials imported from Asian countries, many US manufacturers of herbal products are seeking domestically-produced materials that meet their standards and requirements. Turmeric (*Curcuma longa*) is one such crop. The rhizome from which the curcumins are derived is tuberous, with a rough and segmented skin. The primary rhizome is called the “mother rhizome” or bulb, which is pear-shaped in the center (Figure 1). The branches of mother rhizomes are the secondary rhizomes, called lateral or “finger rhizomes” [11]. The mother rhizomes are more matured than finger rhizomes, therefore containing higher curcuminoid concentrations and perhaps higher essential contents than finger rhizomes. However, the curcumin yield from finger rhizomes is higher than that from mother rhizomes [12].

One of the important components of turmeric is its volatile oil. The role of turmeric oil in the treatment of a wide variety of diseases in animals and humans were reviewed in detail [4,7]. Thus, curcuma oil appears to be a promising agent for the treatment of simple dermatitis, cerebral stroke, and other disorders associated with oxidative stress [13]. The essential oils of turmeric are relatively complex, with hundreds of components. The major components, however, are α-turmerone (12.6–44.5%), β-turmerone (9.1–37.8%), *ar*-turmerone (12.2–36.6%), β-sesquiphellandrene (5.0–14.6%), α-zingiberene (5.0–12.8%), germacrone (10.3–11.1%), terpinolene (10.0–10.2%), *ar*-curcumene (5.5–9.8%), and α-phellandrene (5.0–6.7%) [14].

Turmeric is considered a viable cash crop with a ready market in Alabama and in the US. Similar to any essential oil crop, turmeric’s essential oil also varies with variety, soil type, and environmental conditions [15,16]. Hence, evaluating different varieties for essential content and composition is an important consideration for determining a variety for cultivation in a particular location. Turmeric is a tropical crop and grows well in warm and humid environments with mean air temperatures between 20 and 30 °C. It can be planted in all soil types, but it does best in well-drained clay loam or sandy loam soils rich in humus or organic matter with a soil pH of about 5.5 to 6.5. It grows in a wide range of climatic conditions but requires about 100 to 200 cm of rainfall a year. Thus, Alabama’s hot, humid, and rainy summer season is suited for turmeric production in the southeastern US. Furthermore, turmeric is potentially suited for catering to the herbal products industry, which prefers locally sourced materials. The purpose of the present study was to determine the variation in the essential oil chemistry of five *C. longa* genotypes that could potentially be cultivated for commercial purposes in north Alabama and to note any differences between the mother rhizome and the lateral rhizomes of each cultivar.

## 2. Materials and Methods

### 2.1. Curcuma longa Varieties

The five varieties used in this study were selected out of fourteen varieties according to three criteria: high yield but low curcuminoid content (varieties, CL5, CL3), low yield but high curcumin content (CL10), and high yield and high curcumin content, thus, high curcumin yield (CL9, CL11), based on studies at Auburn University and Alabama A&M University. Thus, CL3 and CL5 may be considered for the fresh rhizome market, CL 10 for the high curcumin dry rhizome herbal products market, and CL9 and CL11, which have attractive, orange-colored rhizomes, could serve both fresh and dry herbal produce markets. The two varieties CL3 and CL9 were consistent in their performance over three years in both south and north Alabama. A knowledge of their relative oil yield and composition could help in value-addition for either fresh rhizomes or dry herbal markets.

### 2.2. Cultivation of Curcuma longa

The rhizomes of five turmeric varieties belonging to *C. longa* (CL3, CL5, CL9, CL10, and CL11) obtained from various sources (Table 1) were planted in seed germination trays filled with a soilless potting mix (Pro-Mix) on 3 April 2019. After planting, the trays were placed in a glass greenhouse at Alabama A&M University, Normal, AL (natural daylight increasing from 11 h in mid-March to about 14.5 h in early June; mean air temperature maintained at 26 °C) for 70 days for sprouting and plant development. The 10-week-old plants were then transplanted onto raised beds (60 cm wide, 15 cm high, 25 m long, 2 m apart, covered with black plastic mulch with drip irrigation tubing underneath the plastic) on 25 June 2019 at the Alabama A&M Winfred Thomas Agricultural Research Station located in Hazelgreen, AL (Latitude 34°89′ N and longitude 86°56′ W). Soil at the experimental site was a Decatur silt loam (fine, kaolinitic, thermic Rhodic Paleudult). The plants were grown using organic production methods and irrigation was provided as and when needed by the drip method. Prior to making the raised beds, the soil was plowed with a rototiller, and a mixture of composted cow manure, poultry litter, and vermicompost was incorporated into the soil at a rate equivalent to 45.5 kg of N/ha. Once the crop was established, a fish emulsion-based organic soluble fertilizer, Neptune’s Harvest™ (Seven Springs Organic Farming and Gardening Supplies, Check, VA, USA), was applied through the irrigation system at 3-week intervals. Three plants from the middle of each row were harvested in mid-February 2020 by digging the plants, separating the rhizomes from the shoot, and washing clean of soil and debris with forced water jets. The mother and lateral rhizomes (Figure 1) were separated and placed in mesh trays and dried of excess water using fans at room temperature. The rhizomes were then placed in a cooler box with ice and carried to the chemistry department at the University of Alabama in Huntsville for oil extraction and profiling.

### 2.3. Essential Oils

The fresh rhizomes, both mother and lateral rhizomes (Figure 1), were chopped and hydrodistilled for 4 h using a Likens–Nickerson apparatus with continuous extraction of the distillate with dichloromethane. Evaporation of the dichloromethane gave pale yellow to yellow rhizome essential oils (Table 1), which were stored at −20 °C until analysis.

### 2.4. Gas Chromatographic–Mass Spectral (GC–MS) Analysis

Gas chromatography–mass spectrometry was carried out as previously described [14]: Shimadzu GCMS-QP2010 Ultra instrument, electron impact (EI) mode (electron energy = 70 eV), scan range = 40–400 atomic mass units, scan rate = 3.0 scans/s, and GC-MS solution software (Shimadzu Scientific Instruments, Columbia, MD, USA); ZB-5 fused silica capillary column, 30 m length, 0.25 mm internal diameter, 0.25 μm film thickness (Phenomenex, Torrance, CA, USA); He carrier gas, head pressure = 552 kPa, flow rate = 1.37 mL/min; injector temperature = 250 °C, ion source temperature = 200 °C, oven temperature program = 50 °C start, increased by 2 °C/min to 260 °C; 7% w/v solutions, 0.1 μL injections, split mode (30:1). Essential oil components were identified based on both their retention indices, which were determined by reference to a homologous *n*-alkane series, and their mass spectral fragmentation patterns available from the databases [17,18,19,20].

### 2.5. Hierarchical Cluster Analysis

Agglomerative hierarchical cluster (AHC) analysis was carried out using the chemical compositions of the *C. longa* rhizome essential oils. The compositions were treated as operational taxonomic units, with the percentages of the 16 most abundant components (α-turmerone, *ar*-turmerone, β-turmerone, α-phellandrene, 1,8-cineole, α-zingiberene, β-sesquiphellandrene, terpinolene, (6*S*,7*R*)-bisabolone, *p*-cymene, zingiberenol, *ar*-curcumene, β-bisabolene, 7-*epi-trans*-sesquisabinene hydrate, limonene, and *ar*-tumerol), using XLSTAT Premium, version 2018.1.1.62926. Euclidean distance was used to determine dissimilarity, and Ward’s method was used to define the clusters.

## 3. Results and Discussion

The fresh mother and lateral rhizomes (Figure 1) were chopped and hydrodistilled to give pale yellow to yellow essential oils in yields ranging from 0.204% to 0.695% (Table 1). Varieties CL5, CL9, and CL11 gave better essential oil yields (0.443–0.659%) than CL3 or CL10 (<0.3%). The total oil content of CL5, CL9, and CL11 were similar to those reported for Indian chemotypes of *C. longa* [21,22]. In CL5, CL9, and CL10, the mother rhizomes had higher oil yields than the lateral rhizomes. A similar trend was reported for curcumin concentration of turmeric varieties grown in south–central AL [12].

**Figure 2:**
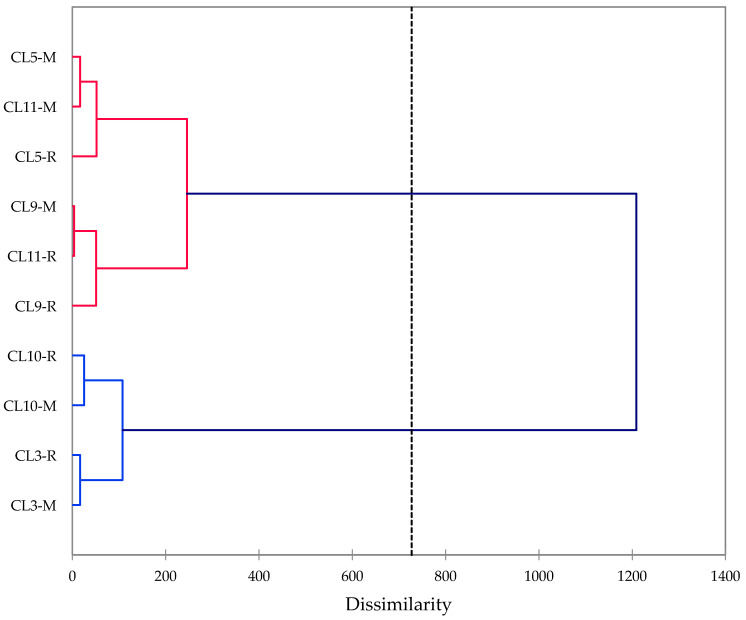
Dendrogram obtained from agglomerative hierarchical cluster analysis of the rhizome essential oils from varieties of *Curcuma longa* cultivated in north Alabama.

The chemical compositions of the *C. longa* rhizome essential oils are compiled in Table 2. The major components in the essential oils were α-phellandrene (3.7–11.8%), 1,8-cineole (2.6–11.7%), α-zingiberene (0.8–12.5%), β-sesquiphellandrene (0.7–8.0%), *ar*-turmerone (6.8–32.5%), α-turmerone (13.6–31.5%), and β-turmerone (4.8–18.4%). A hierarchical cluster analysis of the *C. longa* rhizome essential oils in this study was carried out based on the concentrations of the 16 most abundant essential oil components (α-turmerone, *ar*-turmerone, β-turmerone, α-phellandrene, 1,8-cineole, α-zingiberene, β-sesquiphellandrene, terpinolene, (6*S*,7*R*)-bisabolone, *p*-cymene, zingiberenol, *ar*-curcumene, β-bisabolene, 7-*epi-trans*-sesquisabinene hydrate, limonene, and *ar*-tumerol) (Figure 2).

The cluster analysis of the *C. longa* varieties in this work revealed two well-defined groups. One group (varieties CL5, CL9, and CL11) was dominated by turmerones (α-turmerone, *ar*-turmerone, and β-turmerone). The second group demonstrated lower concentrations of turmerones, but higher concentrations of other components (e.g., α-zingiberene and β-phellandrene). Previous examination of *C. longa* rhizome essential oils from India as well as other geographical locations showed there to be four clusters based on the relative concentrations of the turmerones [14]: (1) dominated by turmerones, but with relatively large concentrations of other components; (2) dominated by turmerones, especially *ar*-turmerone; (3) dominated by turmerones, especially α-turmerone; and (4) very large concentrations of *ar*-turmerone. The chemical compositions of varieties CL5, CL9, and CL11 placed them into the cluster dominated by turmerones (i.e., cluster 2 of [14]), while varieties CL3 and CL10 had relatively lower concentrations of turmerones with relatively larger concentrations of components other than turmerones (i.e., cluster 1 of [14]). Thus, the essential oil compositions of these turmeric varieties adaptable to the Alabama summer growing season fit in well with essential oil compositions of turmeric varieties cultivated in Asia. The *ar*-turmerone and α-turmerone levels were similar to (CL3) or greater than (CL5, CL9, and CL11) those reported for turmeric grown in a tropical country, Brazil [23]. The *ar*-turmerone and α-turmerone were substantially greater than those reported for turmeric grown in India [24]. The β-turmerone levels were generally lower than those reported for turmeric grown in tropical countries [22,23,24], but similar to those reported for turmeric grown in Korea [25]. The cluster analysis also showed very little dissimilarity between the mother rhizome essential oil and the lateral rhizome essential oils for each of the varieties.

Xu and coworkers examined the extracts of 160 samples of *C. longa* from China [26]. Gas chromatographic analysis of the volatiles from the extracts revealed three volatile profile types, while high-performance liquid chromatographic (HPLC) analysis showed three types based on curcuminoid content. Unfortunately, Xu et al. identified only 10 volatile components, whereas 79 components were identified in our essential oil work. Furthermore, percent compositions were not reported and only “representative” chromatograms were presented. Nevertheless, although comparison is tenuous, based on the chromatograms, the volatile profile types identified seem to be analogous to essential oil types in our work. Importantly, volatile profile types “a” and “b” correspond to high-curcuminoid type “B” [26]. Therefore, we conclude that high turmerone concentrations are desirable qualities in turmeric essential oil.

The turmerones are responsible for the turmeric-like odor of *C. longa* [27]. In addition to their pungent scent, turmerones are important, biologically active constituents of *C. longa* rhizome essential oils [28], showing in vitro cytotoxic activities against several human tumor cell lines [29,30,31,32], anti-inflammatory activity through attenuated expression of proinflammatory cytokines [33,34], antibacterial activity against Gram-positive organisms [35], antifungal activity against phytopathogenic [36] and dermatopathogenic [37] fungi, mosquito larvicidal activity against *Anopheles gambiae* [38] and *Culex pipiens* [39], and insecticidal activity against the agricultural pests *Sitophilus zeamais* and *Spodoptera frugiperda* [40].

## 4. Conclusions

The chemical profiles of varieties CL5, CL9, and CL11 tested in this study in north Alabama were comparable to those growing in tropical regions of the world, suggesting that these varieties are suitable for commercialization in this region. However, CL3 and CL10 gave relatively poor essential oil yields and essential oils with lower concentrations of the turmerones. There is a growing market for *Curcuma longa* essential oils, with several varieties showing promise for development in the southeastern United States.

## Figures and Tables

**Table 1:** Hydrodistillation details for *Curcuma longa* rhizome essential oils cultivated in north Alabama.

*C. longa* Cultivar (Rhizome)	Source (Origin) of Rhizome	Mass of Rhizome (g)	Mass of Essential Oil (g)	% Yield	Color
CL3 (mother)	Horizon Herbs, 3350 Cedar Flat Road, Williams, Oregon (Hawaii)	236.2	0.5935	0.251	pale yellow
CL3 (lateral)		268.7	0.6797	0.253	pale yellow
CL5 (mother)	James Simon, Rutgers University, New Jersey (India)	214.0	1.3120	0.613	yellow
CL5 (lateral)		281.0	1.5540	0.553	pale yellow
CL9 (mother)	Lam T. Duong (Dak Lak Province, Vietnam)	207.1	1.3645	0.659	yellow
CL9 (lateral)		188.3	0.8050	0.428	yellow
CL10 (mother)	International farmers Market, Chamblee, Georgia (unknown)	232.0	0.6715	0.289	pale yellow
CL10 (lateral)		282.7	0.5763	0.204	pale yellow
CL11 (mother)	Dr. Anand Yadav, Fort Valley State University, Georgia (unknown)	215.0	1.0760	0.500	pale yellow
CL11 (lateral)		264.6	1.4126	0.534	pale yellow

**Table 2:** Chemical compositions of “mother” and “lateral” rhizome essential oils of *Curcuma longa* varieties growing in north Alabama.

RI_calc_	RI_db_	Compound	CL3-M	CL3-L	CL5-M	CL5-L	CL9-M	CL9-L	CL10-M	CL10-L	CL11-M	CL11-L
924	925	α-Thujene	tr	tr	tr	tr	tr	tr	tr	tr	tr	tr
931	933	α-Pinene	0.1	0.2	0.2	0.3	0.1	0.1	0.3	0.3	0.1	0.1
971	972	Sabinene	0.1	0.1	0.1	tr	tr	tr	0.1	0.1	tr	tr
976	978	β-Pinene	tr	tr	tr	tr	tr	tr	0.1	0.1	tr	tr
988	991	Myrcene	0.4	0.6	0.3	0.4	0.2	0.3	0.6	0.7	0.2	0.2
999	1000	δ-2-Carene	tr	tr	tr	tr	tr	tr	tr	tr	tr	tr
1007	1007	α-Phellandrene	5.9	9.1	7.3	11.8	3.7	6.7	8.7	7.8	4.4	5.8
1009	1009	δ-3-Carene	0.1	0.2	0.1	0.2	0.1	0.1	0.2	0.2	0.1	0.1
1016	1017	α-Terpinene	0.2	0.3	0.1	0.2	0.1	0.1	0.3	0.2	0.2	0.2
1019	1022	*m*-Cymene	---	---	---	---	---	---	---	tr	---	---
1024	1025	*p*-Cymene	0.9	2.2	1.0	1.2	0.5	1.2	1.7	3.0	0.6	0.7
1029	1030	Limonene	0.5	0.7	0.5	0.6	0.3	0.4	0.9	0.8	0.4	0.4
1033	1032	1,8-Cineole	9.2	5.1	6.1	3.5	3.4	2.6	11.7	6.2	8.5	3.6
1034	1034	(*Z*)-β-Ocimene	---	tr	tr	tr	tr	tr	tr	tr	---	---
1045	1045	(*E*)-β-Ocimene	tr	tr	tr	tr	tr	tr	tr	tr	tr	tr
1057	1058	γ-Terpinene	0.3	0.4	0.3	0.5	0.2	0.3	0.4	0.4	0.3	0.3
1069	1069	*cis*-Sabinene hydrate	tr	tr	tr	tr	tr	tr	tr	tr	tr	tr
1085	1086	Terpinolene	4.0	4.4	0.7	0.7	0.5	0.7	3.6	3.0	2.0	1.8
1090	1093	2-Nonanone	0.1	---	---	---	---	---	0.1	---	---	---
1090	1093	*p*-Cymenene	---	tr	tr	tr	tr	tr	---	tr	tr	tr
1099	1099	Linalool	0.1	0.1	tr	tr	tr	tr	0.1	tr	tr	tr
1100	1101	*trans*-Sabinene hydrate	---	---	tr	tr	tr	tr	---	---	tr	tr
1101	1101	2-Nonanol	0.2	0.1	---	---	---	---	0.2	0.2	---	---
1124	1124	*cis-p*-Menth-2-en-1-ol	0.1	0.1	tr	0.1	tr	tr	0.1	0.1	tr	tr
1141	1146	Ipsdienol	---	---	---	---	---	---	---	---	tr	tr
1142	1142	*trans-p*-Menth-2-en-1-ol	0.1	0.1	0.1	0.1	tr	0.1	0.1	0.1	tr	tr
1145	1146	Myrcenone	tr	0.1	0.1	0.1	tr	0.1	---	---	0.1	0.1
1149	1146	*trans*-Limonene oxide	tr	0.3	tr	tr	tr	tr	0.2	0.3	tr	tr
1167	1169	*trans*-β-Terpineol	tr	tr	tr	tr	tr	tr	tr	tr	tr	tr
1170	1170	δ-Terpineol	0.1	0.1	0.1	tr	tr	tr	0.1	0.1	0.1	tr
1171	1165	*iso*-Borneol	tr	tr	---	---	---	---	tr	tr	---	---
1171	1171	*p*-Mentha-1,5-dien-8-ol	---	---	tr	tr	tr	tr	---	---	tr	tr
1173	1173	Borneol	tr	tr	tr	tr	tr	tr	tr	0.1	---	---
1180	1180	Terpinen-4-ol	0.4	0.1	0.2	0.1	0.1	0.1	0.4	0.2	0.3	0.1
1187	1188	*p*-Cymen-8-ol	0.1	0.2	tr	tr	tr	tr	0.1	0.2	0.1	tr
1195	1195	α-Terpineol	0.7	0.3	0.3	0.2	0.2	0.2	0.7	0.4	0.6	0.2
1196	1196	*cis*-Piperitol	tr	tr	tr	tr	tr	tr	tr	tr	tr	tr
1203	1202	*cis*-Sabinol	0.1	0.2	0.1	0.1	tr	0.1	0.2	0.3	0.1	tr
1203	1203	*p*-Cumenol	0.1	0.1	---	---	---	---	---	---	tr	tr
1208	1209	*trans*-Piperitol	tr	tr	tr	tr	tr	tr	tr	tr	tr	tr
1289	1289	Thymol	0.1	0.2	0.2	0.2	0.1	0.2	---	---	0.1	0.1
1292	1293	2-Undecanone	tr	tr	---	---	---	---	tr	tr	---	---
1297	1300	Carvacrol	tr	tr	tr	tr	tr	tr	tr	tr	tr	tr
1309	1312	Livescone	---	---	---	---	---	---	---	---	tr	tr
1319	1318	3-Hydroxycineole	0.1	0.1	tr	tr	tr	tr	0.1	0.3	tr	tr
1400	1405	Sesquithujene	0.1	0.1	---	---	---	---	0.2	0.2	---	---
1417	1417	(*E*)-Caryophyllene	0.1	0.2	0.1	0.2	0.1	0.1	0.1	0.2	0.1	0.2
1431	1432	*trans*-α-Bergamotene	0.1	tr	---	---	---	---	0.1	0.1	---	---
1442	1439	(*Z*)-β-Farnesene	tr	tr	tr	tr	tr	0.1	---	---	0.1	0.1
1450	1452	(*E*)-β-Farnesene	0.3	0.2	tr	tr	tr	tr	0.3	0.3	tr	0.1
1453	1454	α-Humulene	tr	tr	tr	tr	tr	tr	---	---	tr	tr
1476	1482	γ-Curcumene	0.1	tr	tr	tr	tr	tr	0.1	0.1	tr	tr
1480	1482	*ar*-Curcumene	1.1	1.5	0.3	0.3	0.4	0.5	1.9	3.2	0.3	0.5
1482	1483	*trans-*β-Bergamotene	0.1	tr	tr	tr	tr	tr	0.1	0.1	tr	tr
1495	1494	α-Zingiberene	9.2	7.8	0.9	0.9	1.2	0.9	12.5	10.4	0.9	1.0
1507	1508	β-Bisabolene	1.3	1.2	0.2	0.2	0.2	0.2	2.0	2.2	0.2	0.2
1508	1511	β-Curcumene	tr	tr	tr	tr	tr	tr	---	---	tr	tr
1524	1523	β-Sesquiphellandrene	5.5	4.9	0.7	0.8	0.9	0.9	7.7	8.0	0.8	1.0
1526	1528	(*E*)-γ-Bisabolene	0.1	0.2	tr	0.1	tr	tr	0.1	0.1	tr	tr
1553	1555	7-*epi-cis*-Sesquisabinene hydrate	0.4	0.4	0.2	0.2	0.2	0.2	0.5	0.5	0.2	0.2
1559	1561	(*E*)-Nerolidol	0.1	0.1	0.1	0.1	0.1	0.1	0.1	0.1	0.1	0.1
1577	1578	*ar*-Tumerol	0.4	0.5	0.5	0.5	0.6	0.8	0.3	0.3	0.5	0.9
1589	1590	7-*epi-trans*-Sesquisabinene hydrate	0.9	0.8	0.5	0.4	0.5	0.4	1.1	1.2	0.4	0.4
1600	1594	*anti-anti-anti*-Helifolen-12-al B	0.2	0.2	0.3	0.4	0.4	0.4	0.2	0.2	0.2	0.4
1615	1615	Zingiberenol	2.0	1.7	0.3	0.5	0.6	0.5	2.8	3.0	0.5	0.6
1620	1620	*anti-syn-syn*-Helifolen-12-al C	0.2	0.3	0.3	0.3	0.5	0.3	---	---	---	0.6
1623	1624	10-*epi*-γ-Eudesmol	0.2	0.2	---	0.2	---	0.4	0.2	0.4	---	0.1
1643	1647	Camphenone	---	---	---	---	---	---	---	0.3	---	---
1670	1668	*ar*-Turmerone	15.4	15.3	18.3	15.5	26.3	32.5	6.8	9.5	21.8	27.0
1675	1668	α-Turmerone	17.6	18.7	30.1	31.5	24.9	18.9	15.4	13.6	27.8	24.8
1687	1687	Himachal-4-en-1β-ol	0.7	0.7	---	---	---	---	0.9	1.0	---	---
1688	1687	α-Bisabolol	0.5	0.3	---	---	---	---	0.5	0.8	---	---
1695	1695	(2*Z*,6*Z*)-Farnesol	0.2	0.3	---	---	---	---	0.4	0.6	---	---
1702	1699	β-Turmerone (= Curlone)	8.9	8.8	17.1	16.8	18.4	17.0	5.0	4.8	17.9	17.5
1712	1712	Curcuphenol	0.2	0.2	0.2	0.2	0.2	0.2	0.2	0.2	0.1	0.2
1743	1742	(6*S*,7*R*)-Bisabolone	2.0	1.7	0.6	0.7	0.8	0.7	3.1	3.4	0.7	0.7
1773	1775	*trans*-α-Atlantone	0.3	0.2	0.2	0.4	0.4	0.4	0.2	0.2	0.3	0.4
1807	1807	Eudesm-11-en-4α,6α-diol	0.4	0.4	---	---	---	---	---	---	---	---
1985	1983	Methyl-β-(*E*)-ionyl tiglate	0.5	0.4	0.1	0.1	0.1	0.2	0.2	0.5	0.1	0.1
		Monoterpene hydrocarbons	12.6	18.3	10.6	15.9	5.6	9.9	17.0	16.6	8.3	9.5
		Oxygenated monoterpenoids	2.0	2.0	0.9	0.8	0.5	0.7	2.1	1.9	1.4	0.6
		Sesquiterpene hydrocarbons	17.9	16.1	2.2	2.5	2.8	2.7	25.0	24.8	2.3	2.9
		Oxygenated sesquiterpenoids	51.1	51.1	68.9	67.8	74.0	73.0	37.9	40.5	70.5	73.8
		Others	0.3	0.1	0.0	0.0	0.0	0.0	0.3	0.2	0.0	0.0
		Total Identified	83.8	87.6	82.7	86.9	82.9	86.2	82.2	84.0	82.4	86.8

RI_calc_: Retention indices determined with respect to a homologous series of *n*-alkanes on a ZB-5 column. RI_db_: Retention indices from databases [17,18,19,20]. tr: “trace” (<0.05%).

## Data Availability

Data are available from the corresponding authors.
